# Size, not color, drives assortative mating and influences fledging survival, weight and immunity in a polymorphic owl

**DOI:** 10.1038/s41598-025-04191-1

**Published:** 2025-06-02

**Authors:** Deseada Parejo, Erick González-Medina, Ángel Cruz-Miralles, Jesús Miguel Avilés

**Affiliations:** 1 Departamento de Ecología Funcional y Evolutiva, EEZA-CSIC, La Cañada de San Urbano, Almería Spain; 2 Unidad Asociada (CSIC-UEX): Ecología en el Antropoceno, Facultad de Ciencias, Universidad de Extremadura, Badajoz, Spain; 3 Grupo de Investigación en Conservación. Facultad de Ciencias., Universidad de Extremadura, Badajoz, Spain; 4 Departamento de Biodiversidad, Ecología y Evolución, Facultad de Ciencias Biológicas, Universidad Complutense, Madrid, Spain

**Keywords:** Body size, Color polymorphism, Non-random mating, Owls, Reversed sexual size dimorphism, Ecology, Evolution, Zoology

## Abstract

The persistence of color polymorphism in nature may be driven by disassortative mating based on color. In vertebrates, body size sometimes correlates with coloration, complicating mating patterns, as the selective pressures favoring mixed-color pairs might be counterbalanced by those influencing body size. This complexity is heightened in species with reversed sexual size dimorphism, such as owls, where males are smaller than females, and average dissimilarity in mate size may reflect sexual size dimorphism rather than an active disassortative mating pattern. Here we investigate the fitness consequences of mating by color and body size using a long-term dataset from the color polymorphic Eurasian Scops owl (*Otus scops*), a bird species with reversed sexual size dimorphism. Results reveal that size-disassortative mating enhances reproductive success, as highly size-dimorphic pairs have higher probability of fledging owlets, which may favor reversed sexual size dimorphism. In addition, larger pairs produce heavier owlets with higher immunocompetence, aligning with the conventional size-based mating hypothesis. Although body size and plumage coloration were correlated within pairs, only differences in body size between pair members, not coloration, were related to higher fitness estimates. While color-based assortative mating had no direct impact on any of the fitness proxies studied, greyer pairs exhibited higher feeding rates to offspring than browner pairs. These results underscore the importance of simultaneously considering traits that may covary with color and shape mating patterns to understand the persistence of color polymorphisms in nature.

**Supplementary Information:**

The online version contains supplementary material available at 10.1038/s41598-025-04191-1.

## Introduction

Understanding how color polymorphisms persist in nature is intriguing because, although numerous hypotheses have been proposed, no single mechanism universally explains all cases^1,2^. This question is pivotal to explain the evolution of genetically determined phenotypic diversity, given that color variants are primarily genetically determined^3,4^. Disassortative mating, wherein individuals with distinct phenotypes are more likely to mate, is a rare form of non-random mating pattern that has been proposed as one of the mechanisms that could explain the maintenance of polymorphisms^5–8^. This mating pattern promotes the production of heterozygous offspring^9,10^, which could help to maintain genetic diversity when diverse or heterozygous young are favoured. In the context of color polymorphisms, selection may favor disassortative mating if pairing with a partner of a different color morph confers fitness advantages, particularly in terms of prey delivery to offspring. Mixed-morph pairs may exploit distinct ecological niches^11–13^, leading to more stable feeding rate for their intermediate offspring^14^. This idea, known as the “complementarity hypothesis”^2^, suggests that such pairing, therefore, enhances offspring survival. Support for this hypothesis comes from black sparrowhawks (*Accipiter melanoleucus*) where mixed-color pairs deliver more food to offspring^15^, leading to produce annually more offspring^14^ with higher survival rates^16^, though with lower body condition^14^, compared to similar-color pairs. Alternatively, the advantage of disassortative mating could arise through an increase in genetic variability in the offspring due to heterozygosis, which can enhance genetic variability and potentially improve adaptability to environmental changes, depending on the traits and genes involved. This mechanism has been proposed, with varying degrees of evidence, as a potential explanation for the maintenance of color polymorphism across multiple taxa, including butterflies (*Heliconius numata)*^8^, grasshoppers (*Tetrix subulata* and *Tetrix undulata)*^17^, birds such as the common buzzard (*Buteo buteo)*^18^,but see^19^, white-throated sparrow (*Zonotrichia albicollis)*^20^ and common guillemot (*Uria aalge*)^21^, as well as mammals like the wolf (*Canis lupus*)^22^. In Yellowstone, black and grey wolves mate disassortatively^7^, mainly producing black heterozygote cubs which exhibit higher fitness^23^.

However, disassortative mating is rare in nature^6,24^, particularly when it is based on color^[but see 25^, and has only been occasionally observed in color-polymorphic animals^[e.g. 7,26–28^. The context-dependent adaptation of particular phenotypes to specific environmental or social conditions could explain why, in those species, disassortative mating based on coloration is not observed. For instance, female pygmy swordtails (*Xiphophorus pygmaeus*) prefer blue males over dominant, more aggressive gold males only in populations where predation risk is low. Under high predation risk, however, females show no preference for blue males^29^. Similarly, in the color polymorphic Eleonora’s falcon (*Falco eleonora*), mating seems to be random regarding coloration, likely because the success of the different color pairs depends on environmental conditions^30^. Alternatively, random mating based on coloration might arise when mate preferences are driven by a suite of traits that covary with coloration. Indeed, the expression of color morphs is often linked to other phenotypic traits^31^. For example, coloration may covary with body size^32^, a trait that can also influence mate selection^33^.

The presence of size-assortative mating in color polymorphic species with sexual dimorphism in size may influence color-based mate choice, especially if coloration and body size covary or are perceived jointly during mate selection. Sexual dimorphism in size occurs in certain vertebrate orders such as Strigiformes (class Aves), where 33.5% of species are color polymorphic^34^. Notably, all species in this clade display reverse sexual size dimorphism, with females being larger than males^35^. Conventional expectations of size-based assortative mating assume a preference for larger, more fecund females and larger, more competitive males^36^. This expectation is challenged in species with reversed sexual size dimorphism^37–39^. In such species, average dissimilarity in mate size may be observed, which may reflect sexual dimorphism rather than an active disassortative mating pattern. In owls, the evolution of reversed sexual size dimorphism seem to be mainly shaped by natural selection favoring smaller males due to their increased agility, maneuverability, and foraging efficiency^37,40^. As a result, mating pairs often consist of a relatively small male and a larger female^41^. Therefore, the simultaneous study of both color- and size-based mating and their fitness consequences in color polymorphic animals seems critical for a comprehensive understanding of how natural and sexual selection shapes phenotypic variation within populations. To our knowledge, this fundamental question has been addressed in only five vertebrate species: the red-backed salamander (*Plethodon cinereus*)^42,43^, the strawberry poison-dart frog (*Oophaga pumilio*)^44^, the common wall lizard (*Podarcis muralis*)^45^, the Arctic jaeger (*Stercorarias parasiticus*)^46^ and the feral pigeon (*Columba livia*)^27^. Notably, only two of these studies (on the common wall lizard and the red-backed salamander) examined cases involving reversed sexual size dimorphism. In a wild population of the common wall lizard, for instance, where there was no covariation between coloration and body size, researchers found some evidence of color assortative pairing, but not of size assortative mating^45^. In the red-backed salamander, morph-dependent mating patterns co-occur with size-related pairing, as larger males showed a preference for more fecund, larger females. This suggests that factors other than mate choice may drive the evolution of color polymorphism evolution in the species^42,43^. However, none of these studies focused on avian species. This highlights the novelty of our approach, both for vertebrates in general and for birds in particular.

Here, we analyze a long-term (2012–2021) dataset from a breeding population of the Scops owl (*Otus scops*), a sexually dimorphic and color polymorphic Strigiform, to investigate the mating pattern based on coloration and body size, as well as their consequences in terms of fitness. In this species, males are smaller than females and individuals can be categorized into three discrete color morphs (grey, intermediate and brown)^47^, although its plumage coloration varies continuously from grey to brown in relation to the amount of phaeomelanin^48^. Moreover, in the study population we found a relationship between coloration, territoriality, and stress response in males; however, the relationship between these traits and body size was not studied^49^. As we hypothesize that fitness consequences of a pairing based on coloration may depend on body size, firstly, we determined the degree of size-color covariation in the species in the studied population. Plumage coloration and body size are positively related in both males and females (see results, Figure S1), so that bigger individuals were browner, and, hence, we made predictions considering this covariation under three different hypothetical mating scenarios (Table 1):

**Table 1:** Predictions emerging from the different hypotheses proposed to explain the fitness of different parental combinations in the Scops owl and observed results supporting them.

Mating scenario	Parental combinations with the highest fitness	Observed results
Color-based mating	Dissimilar pairs in color	NO
Conventional size-based mating	Larger pairs	✔ Larger pairs produced offspring in better condition
Size-based mating under the theory of reversed sexual size dimorphism	Larger females + smaller males	✔ Pairs with large females and small males showed higher reproductive success

1. Color-based mating: We predict that dissimilar scops owls’ pairs in coloration will show improved reproductive outcomes. Given that coloration positively correlates with body size in this population, we also expect that dissimilar pairs in size will achieve better reproductive success and produce offspring in better condition than more similar pairs.
2. Conventional size-based mating: We predict that larger pairs will achieve greater reproductive success and raise chicks in better condition than smaller pairs. Given the observed covariation between coloration and body size, we also expect that browner pairs will have more favourable reproductive outcomes.
3. Size-based mating under the theory of reversed sexual size dimorphism: We predict that the most successful pairs will be those with larger females and smaller males because each partner independently possesses traits enhancing reproductive success (larger female size associated with increased fecundity and incubation efficiency, and smaller male size linked to enhanced foraging efficiency, maneuverability, and agility). Moreover, based on the covariation between coloration and body size, we also expect that the pairs with browner females and greyer males will achieve greater reproductive success and produce chicks in better condition.

Finally, we assess whether pair assortment in color and size affects foraging effort and prey delivered to offspring. This analysis aims to evaluate whether differences in feeding efficiency could result from specific mating combinations, thereby providing a potential functional explanation for the observed mating patterns. In agreement with the “Complementarity Hypothesis”, pairing with a dissimilar partner (in color or size) may improve feeding efficiency^[e.g. 15^.

## Results

Morphometric measurements revealed significant sexual dimorphism in Scops owls, with females being consistently larger than males across all measured traits (Table S1). The most pronounced difference was observed in body mass, where females weighed significantly more than males (mean = 97.61 g vs. 69.63 g; t = 26.21, df = 219, *p* < 0.0001). Besides, females had significantly greater wing length (t = 8.02, df = 219, *p* < 0.0001), bill length (t = 3.53, df = 220, *p* = 0.0005) and tarsus length (t = 2.69, df = 219, *p* = 0.008) than males (Table S1). However, coloration did not differ between males and females, indicating that there is no sexual dichromatism (t = -0.29, df = 215, *p* = 0.77). Additionally, a positive correlation was observed between wing length (the proxy for body size used in our analyses, see methods) and plumage coloration, irrespective of sex (Figure S1, Color morph score effect: F_1,212_ = 6.78, *P* = 0.01, Estimate ± SE = 0.60 ± 0.30; Interaction Color morph score*Sex: F_1,212_ = 0.12, *P* = 0.72), with browner individuals tending to have longer wings (Figure S1).

### Assortative mating

**Fig. 1:**
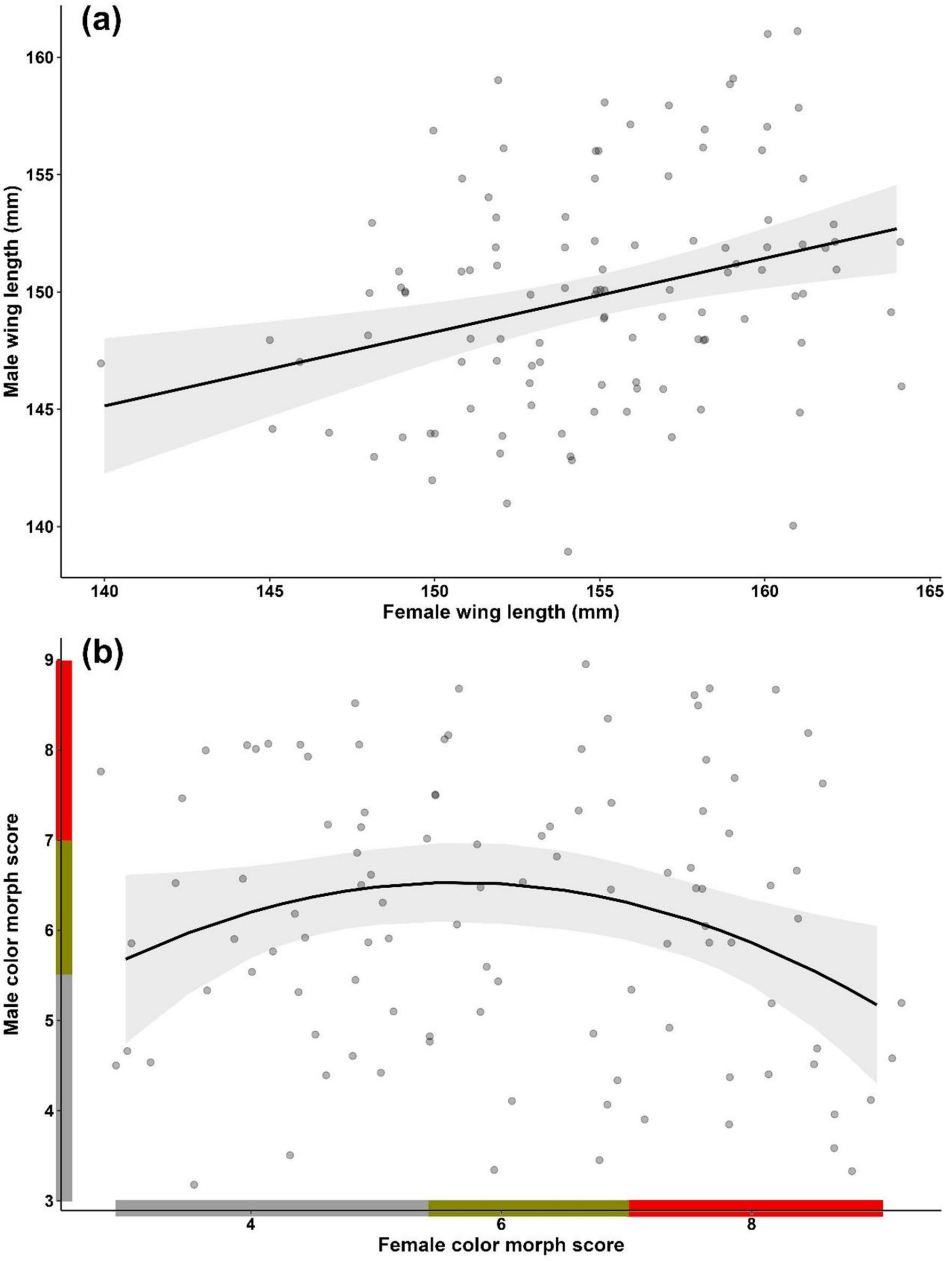
Relationship between (**a**) wing length of female and male, and (**b**) color morph score of female and male Scops owls (*Otus scops*) during the breeding season. Colors on axes of (**b**) indicate the color morph of individuals as grey (scores < 5.5), intermediate (scores between 5.5 and 7, both included), and brown (scores > 7). Figures show the predicted values (lines) derived from the linear models presented in Table S2. The shaded areas represent the 95% confidence intervals of the fitted models. In addition, uncorrected raw data is shown as black transparent dots in the background.

Significant positive relationships were found between pair members for wing length (F_107,109_ = 13.22, *p* < 0.001, Table S2) and color morph (linear term: F_102,105_ = 4.99, *p* = 0.03; quadratic term: F_102,105_ = 5.67, *p* = 0.02, Table S2), controlling for the relative age of the female. Larger females tended to pair with larger males (Fig. 1a), while females with the greyest or brownest plumages showed a preference for grey males (Fig. 1b).

### Consequences of assortative mating on fitness proxies

**Fig. 2:**
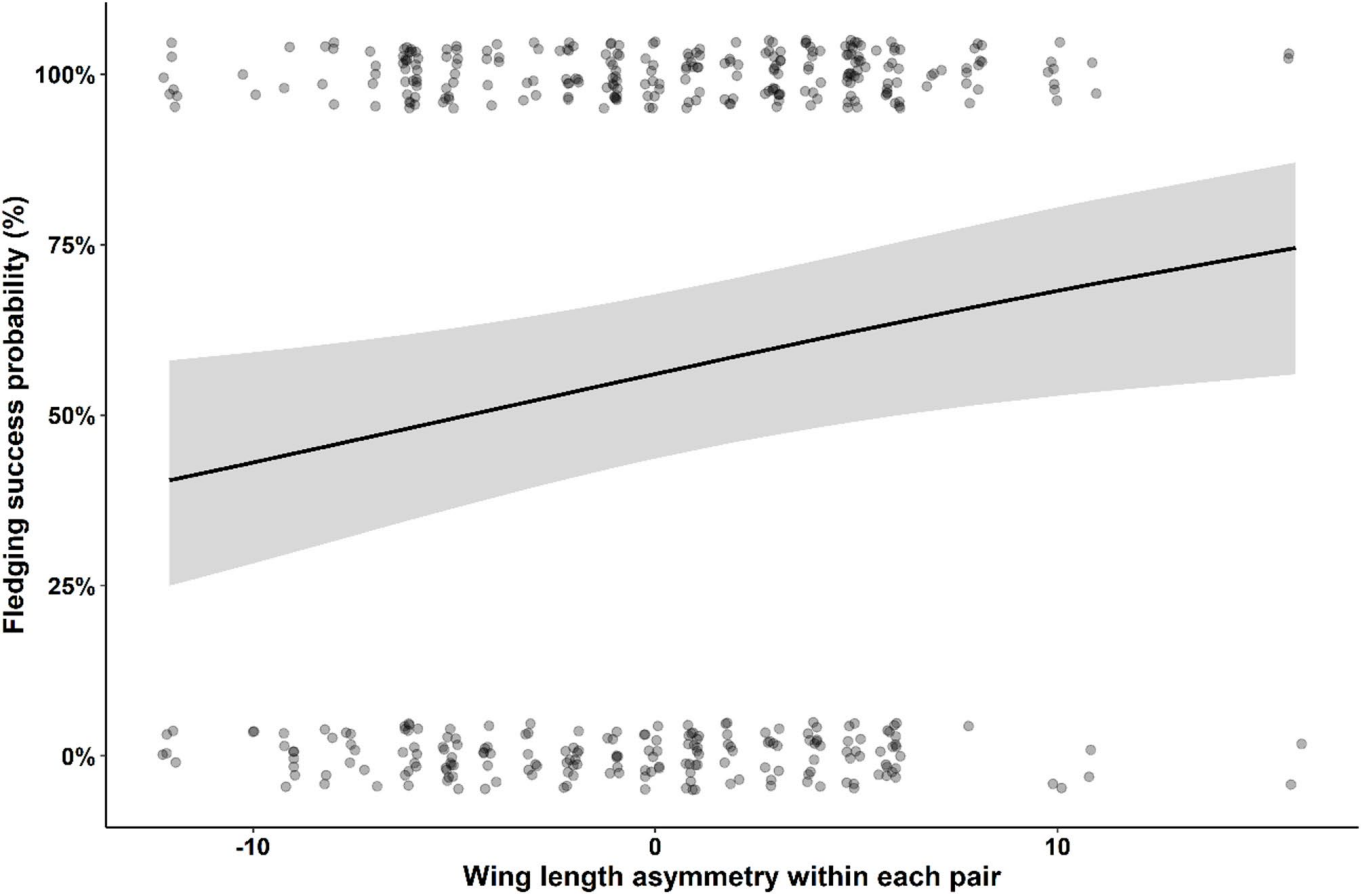
Relationship between fledgling success probability of each chick and the asymmetry in wing length within each pair. In the X axis values near 0 indicate typical pairs with reversed sexual size dimorphism, negative values indicate pairs with relatively similar-sized individuals, and positive values indicate highly dimorphic pairs, with larger than average females and smaller than average males. Figure shows the predicted values (lines) derived from a generalized linear mixed model presented in Table S3. The shaded areas represent the 95% confidence intervals of the fitted models. In addition, uncorrected raw data is shown as black transparent dots in the background.

Fledging success was positively related to size asymmetry between pair members (fledging success: Estimate ± SE = -0.05 ± 0.022, F_1_,_330_ = 5.28, *p* = 0.02; Fig. 2), but not to the overall size of the pair (Table 2). In contrast, neither the number of fledglings nor fledging success were significantly influenced by assortative mating in coloration nor by the total coloration of the pair (Table 2).

**Table 2:** Effect of overall pair value (Sum color and sum wing) and asymmetry (Dif color and res wing) in wing length and color score within each pair on number of fledglings per nest, fledging success of fledglings in the Scops owl (*Otus scops*). Bold values indicate P values < 0.10.

Predictors	Number of fledglings per nest*				Owlet’s fledging success				Owlets’ body-mass at fledging				Owlets’ PHA response			
	Estimate	Std. error	t-value	p	Estimate	Std. error	t-value	p	Estimate	Std. error	t-value	p	Estimate	Std. error	t-value	P
(Intercept)	0.65	2.81	0.23	0.82	3.93	4.88	0.81	0.44	12.26	34.87	0.35	0.73	− 1.87	1.08	− 1.74	0.13
Sum color	− 0.003	0.03	− 0.11	0.91	0.02	0.06	0.36	0.72	− 0.65	0.39	− 1.66	0.1	0.01	0.01	1.07	0.29
Dif color	− 0.01	0.05	− 0.27	0.79	− 0.05	0.08	− 0.59	0.56	− 0.28	0.63	− 0.45	0.65	− 0.01	0.02	− 0.38	0.7
Sum wing	0.001	0.01	0.08	0.93	− 0.09	0.16	− 0.54	0.59	**2.09**	**1.16**	**1.8**	**0.07**	**0.07**	**0.03**	**1.94**	**0.05**
Res wing	0.02	0.01	1.51	0.13	**0.05**	**0.02**	**2.3**	**0.02**	0.02	0.15	0.15	0.88	0.01	0.004	1.42	0.16
Brood size	–	–	–	–	**-0.27**	**0.16**	**− 1.72**	**0.09**	0.07	1.04	0.07	0.94	− 0.02	0.03	− 0.52	0.6
Random effects																
τ_00 Nest_	–				0.12				15.93				0.03			
τ_00 Year_	0.07				0.56				27.16				0.06			
N_Year_	10				10				10				8			
N_Nest_	–				105				97				83			
Observations	105				437				243				184			

The magnitude of overdispersion (measured as the ratio of the Pearson *χ*2 value to its corresponding *88 degrees of freedom *n* – *p*) was *0.53 in the model with the Poisson error distribution, suggesting some subdispersion.

**Fig. 3:**
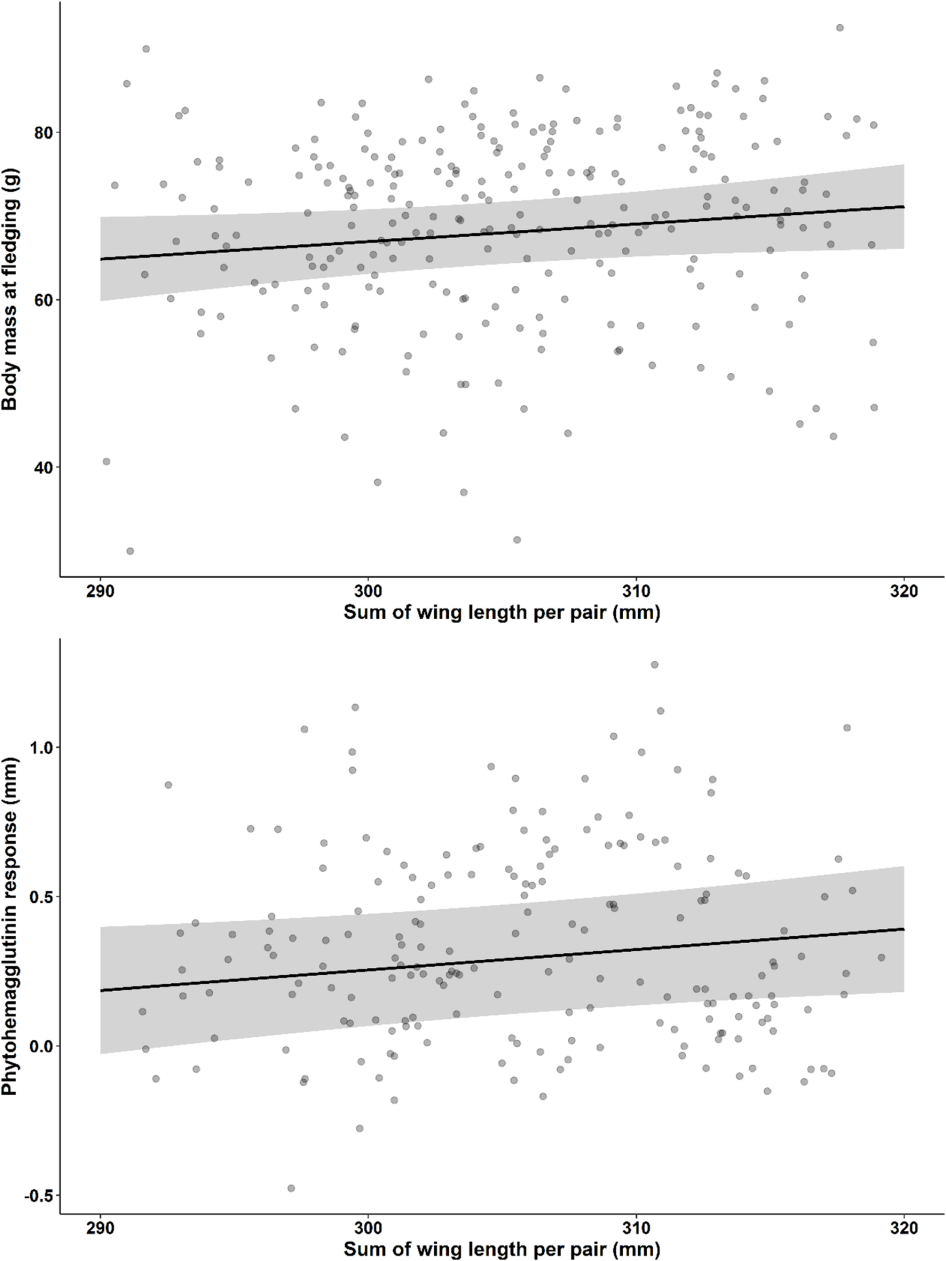
Relationship between overall pair value in wing length (sum of wing length per pair) in Scops owls (*Otus scops*) and owlets’ (**a**) body-mass, and (**b**) phytohemagglutinin response, at fledging. Figures show the predicted values (lines) derived from linear mixed models presented in Table S4. The shaded areas represent the 95% confidence intervals of the fitted models. In addition, uncorrected raw data is shown as black transparent dots in the background.

We also found that larger pairs had heavier owlets at fledging (Estimate ± SE = 2.09 ± 1.16, t_146_ = 1.80, *p* = 0.07; Fig. 3a), although this result was only marginally significant, and with higher PHA response (Estimate ± SE = 0.07 ± 0.03, t_104_ = 1.94, *p* = 0.05; Fig. 3b) (Table 2). However, the sum of color scores from both parents was not related with owlet body mass or PHA response (Table 2). Additionally, neither asymmetry of wing length nor color were related to these condition estimators of the owlets (Table 2).

### Feeding efficiency in relation to size and color of partners

**Fig. 4:**
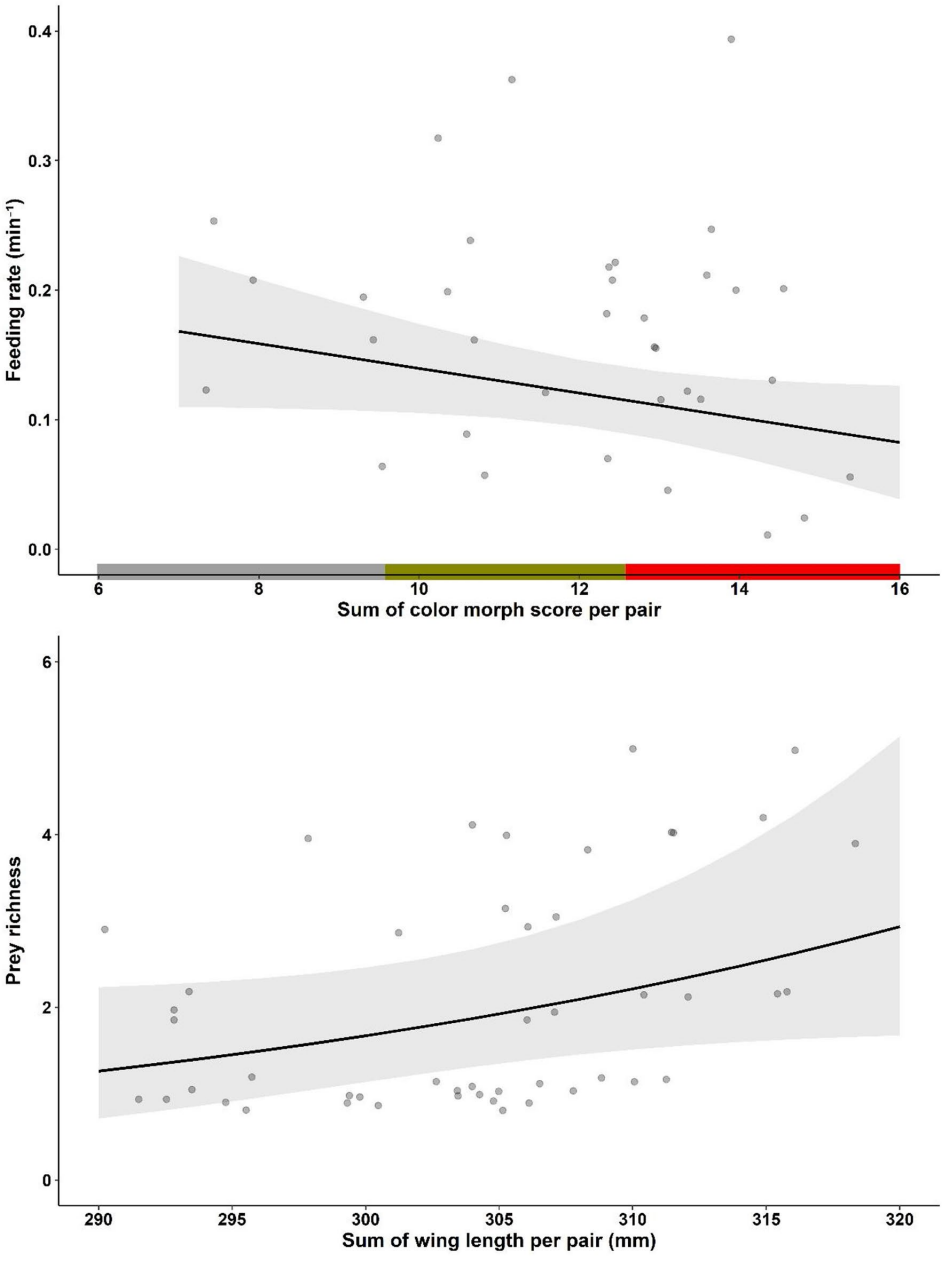
Relationship between (**a**) overall pair value in color morph (sum of color morph scores per pair) in Scops owls (*Otus scops*) and feeding rates (min^− 1^) and (**b**) overall pair value in wing length (sum of wing length per pair) and prey richness at nests. Figures show the predicted values (lines) from linear mixed models presented in Table S5. The shaded areas represent the 95% confidence intervals of the fitted models. In addition, uncorrected raw data is shown as black transparent dots in the background.

Prey richness per nest was marginally related to the overall size of the pair, such that larger pairs tended to provide a more variable array of prey to their offspring compared to smaller pairs (Estimate ± SE = 0.03 ± 0.01, t_41_ = 1.80, *p* = 0.08, Table 3; Fig. 4). Neither the degree of assortative mating based on pair size or coloration, nor the overall coloration of the pair, was related to the prey richness delivered at nests (Table 3). However, feeding rates per nest was marginally negatively related to overall pair coloration and pairs with browner plumage fed less frequently compared to those with greyer plumage (Estimate ± SE = -0.01 ± 0.005, t_44_ = -1.96, *p* = 0.06, Table 3; Fig. 4). None of the other independent variables were related to the feeding rate per nest (Table 3).

**Table 3:** Effect of overall pair value (Sum color and sum wing) and asymmetry (Dif color and res wing) in wing length and color score within each pair on feeding rates and prey richness per nest. Bold values indicate P values < 0.10.

Predictors	Feeding rate				Prey richness*			
	Estimate	Std. error	t-value	p	Estimate	Std. error	t-value	p
(Intercept)	0.07	0.42	0.17	0.87	− 7.04	4.43	− 1.59	0.21
Sum color	**− 0.01**	**0.005**	**− 1.96**	**0.06**	− 0.05	0.05	− 1.01	0.32
Dif color	− 0.002	0.01	− 0.29	0.78	0.03	0.08	0.44	0.66
Sum wing	0	0.001	0.4	0.69	**0.03**	**0.01**	**1.8**	**0.08**
Res wing	0.002	0.002	0.71	0.48	− 0.01	0.02	− 0.26	0.79
Random effects								
τ_00 Year_	0				0.12			
N_Year_	6				4			
Observations	54				49			

The magnitude of overdispersion (measured as the ratio of the Pearson *χ*2 value to its corresponding *41 degrees of freedom *n* – *p*) was *0.40 in the model with the Poisson error distribution, suggesting some subdispersion.

## Discussion

Our study on the Eurasian scops owl in Spain contributes to our understanding of the evolutionary implications of assortative mating across multiple traits in color polymorphic animals by testing several hypotheses. We found significant relationships between male and female wing length and plumage coloration across pairs. Regardless of the underlying mechanism driving these associations, pairs with dissimilar wing lengths exhibited higher reproductive success, as predicted by the theory of reversed sexual size dimorphism (Table 1). In addition, pairs with larger wings reared heavier offspring with better inmunity, aligning with the conventional size-based mating hypothesis (Table 1), even for a species like the one under study, which exhibit reversed sexual size dimorphism. However, asymmetry in plumage coloration within mated pairs did not account for differences in reproductive success, despite a positive correlation between wing length and plumage coloration in both sexes. These findings underscore how assortative mating based on multiple traits, including body size and coloration, can influence reproductive outcomes and may play a role for the persistence of color polymorphisms in natural populations.

### The importance of body size

The observed size-assortative pairing in scops owls aligns with broader patterns in birds, where size compatibility is known to enhance reproductive success through improved coordination and resource allocation between parents^6,10,41,50,51^. However, we found a positive relationship between size asymmetry and fledging success, suggesting that dissimilar pairs are more efficient in some parental duties, leading to better offspring outcomes^51,52^. Here, it remains uncertain whether the observed reproductive benefits in size-dissimilar pairs stem directly from enhanced parental resource provisioning because asymmetry in wing length did not explain nocturnal feeding rates or prey richness delivered per nest. However, only nocturnal feeding was recorded in this study and a significant part of feeding activity occurs during the day in this species^13^, when potential foraging advantages associated with body size may become apparent. The size-based mating hypothesis within the framework of reversed sexual size dimorphism proposes that smaller males may exhibit increased agility, enhancing their foraging efficiency and ability to evade predators^37,38^. In this regard, we found that greyer pairs (including greyer males), which are probably smaller in the basis of the covariation color-size, fed their offspring more frequently than browner pairs. Alternatively, the observed reproductive benefits in size-dissimilar pairs might be due to improved nest defense, which, unfortunately, cannot be verified here due to the lack of data. On the other hand, larger pairs reared heavier owlets, although with a marginal significance, with better inmunity. These last findings are consistent with research on other owl species, such as the Ryukyu Scops owl (*Otus elegans*), where larger males have been shown to achieve improved offspring outcomes^41^. Moreover, larger scops owls pairs showed a trend to provide more variable prey to nests compared to smaller pairs, which might be behind the production of owlets in better condition by larger pairs. Our results might be tentatively suggesting that selection is consistently favoring larger females, who would benefit both from be mated with very small males in terms of the number of owlets produced and from be mated with larger males in terms of the owlets’ condition at fledging. On the other hand, selection seems to favor larger males, who produce owlets in good condition, as well as smaller males, who produce more owlets. This potential balance between what could be attributed to foraging efficiency and predator avoidance suggests that both opposing selective pressures may coexist in the mating dynamics of scops owls.

### The importance of plumage color

Our results did not support the hypothesis that reproductive outcomes are determined by color-based assortative mating, as fitness proxies were not related to the asymmetry in parental plumage coloration (Table 1), despite a correlation between size and color in our population. The lack of relationship between reproductive success and pair-color dissimilarity contrasts with findings in other species, where color morph complementarity enhances fitness^14,15^. However, we did observe that nocturnal feeding efficiency varied with overall pair coloration, with browner pairs feeding less frequently than greyer pairs, suggesting that plumage coloration may be linked to ecological roles, potentially reflecting differential foraging success under varying conditions. Previous studies on this species have indeed shown that greyer individuals may be better adapted than browner ones to exploit nocturnal conditions due to their poorer crypsis under daylight^13^, which might potentially explain the observed relationship between plumage coloration and nocturnal feeding efficiency. Hence, different plumage coloration might confer distinct fitness advantages under varied environmental conditions, constituting selective pressures that might either complement or counteract those associated with body size.

### The maintenance of color polymorphism

Our findings contributes to our understanding of the persistence of color polymorphism in the Eurasian scops owl, emphasizing the mediating role of body size in reproductive success. The found positive association between size-disassortative mating and fledging success and the positive relationships between overall pair size and owlet condition at fledging suggest that parental body size complementarity and compatibility, rather than color-based pairing, may be crucial in driving different reproductive outcomes. In the scops owl, natural selection appears to act on body size, favoring large females, likely to be browner due to the observed correlation between size and color, and the smallest and largest males, likely to be the greyest and brownest. The covariation size- color may have implications for the persistence of color polymorphism, as selection on one trait may drive evolutionary changes in the other. Indeed, color polymorphism in the species might be maintained through a dual mechanism: (1) Sexual selection may favor greyer individuals, as both grey and brown individuals predominantly form pairs with grey partners (Fig. 1b). In addition, natural selection may favor smaller, greyer males because, by mating with larger females, they produce more owlets and because greyer pairs show higher nocturnal feeding rates to nests (Fig. 4a), although color-based mating had no direct impact on reproductive outcomes. (2) Natural selection may favor larger, browner individuals, which experience stronger fecundity selection (Figs. 2 and 3). This dual mechanism aligns with broader theories on the maintenance of phenotypic diversity, suggesting that size and coloration may confer adaptive advantages under varying environmental conditions^10,53,54^. Our findings underscore the importance of considering multiple, likely correlated traits, such as color and body size, to better understand the maintenance of color polymorphisms. By revealing how these traits interact to influence reproductive outcomes, our study offers new insights into the evolutionary processes sustaining phenotypic diversity.

## Methods

The study was conducted from 2012 to 2021 in the surroundings of Baza Natural Park in Granada (37°18′N, 3°11′W), southeastern Spain. The area is an extensive agricultural landscape, approximately 22 km^2^ in size, characterized by a low density of natural holes^55^. Within this landscape, 20–35 pairs of scops owl breed annually in cork-made nest-boxes attached to holm oak trees *Quercus ilex*^56,57^, with an average density throughout the study years of 1.24 pairs/km^2^.

Scops owls are medium-sized nocturnal and trans-Saharan migratory birds that arrive to the study area throughout April from their African winter quarters^56,58^. The species shows reversed sexual size dimorphism, with females being heavier and with longer wings than males^59^. They start breeding in May^56^, typically laying a clutch of about 2 to 6 eggs annually. Incubation is carried out by the female, begins from the laying of the second egg and takes 24–25 d^60^. Both sexes participate in feeding tasks, although just after hatching, females are mainly brooding owlets and males are mainly in charge of hunting^60^.

### Field data collection

Every year, nest boxes were visited once a week until egg-laying was detected. After occupation and before hatching, the nests were revisited only once more after the end of the laying and just before the estimated hatching date to capture and ring the incubating female by hand (while sleeping during the day). After hatching, nests were monitored weekly to record brood size and the number of fledglings. Males were captured with nest-traps at night during the chick-rearing period while they delivered food to the nests. Adult sex determination was based on inspection of the brood patch (only present in females), and was verified through genetic sex determination (authors, unpublished data).

Upon capture, all adults were measured (wing length, bill and tarsus length (precision of 0.01 mm), and weight (precision of 0.5 g)), ringed for individual identification in subsequent years, and photographed for color assignment^[as in 47^. We took two standardized photos of each captured individual: one head-on to capture head and breast plumage, and one from the back to capture the back and wings. Photos were taken with a Canon EOS 1300D camera (Lens EF-S 18–55 IS II) mounted on a tripod at a constant 50 cm distance, using a flash (aperture 4.5, shutter speed 1/200, ISO 800). Owls were gently secured in a carton box with stable lighting, with their heads placed next to a color chart (X-Rite ColorChecker^®^ Passport). Photos were standardized using Adobe^®^ Photoshop Lightroom 6 and analyzed for redness extension in the head, breast, and wings-back. Each body part was scored by eye from 1 to 3 based on its predominance of grey or brown, with total scores ranging from 3 to 9. Birds were classified as grey (scores < 5.5), intermediate (5.5–7), or brown (> 7) following the trimodal distribution of scores^47^. This scoring method was repeatable among researchers (F_27,28_=11.054; *p* < 0.001; R^2^ = 0.78) and validated through spectrophotometric analysis of feathers from 129 individuals, confirming its reliability in assessing body redness in scops owls^47^.

In the study area, most local fledglings that returned to breed (87.5% of individuals) were at least 2-years-old. Hence, for every bird of unknown origin and age recruited into the population, we assigned a minimum age of 2 years at the time of first capture and calculated their relative age from that point onward. Therefore, the age assigned to all immigrants into our population during their first capture was 2 years, and one year was added for each subsequent year.

In each nest, at the end of the nesting period, we collected some data to estimate the fitness associated to different parental combinations. Specifically, we recorded the number of fledglings and weighed fledglings to the nearest 0.50 g using a Pesola spring balance. Moreover, when the youngest chick in each nest reached 18 days-old, all owlets were injected subcutaneously with phytohemagglutinin-P (PHA-P; Sigma Chemical) in the wing web to evaluate the in vivo T cell-mediated immune response replicating previously described protocols^61,62^.

Feeding behavior was filmed at nests after dawn with infrared cameras (KPC- S500, black and white CCD camera, Essentia Systems Inc.) located inside nest boxes in the first third of the nesting period (8 days after the hatching of the first egg). Recordings lasted 73.98 min on average (± 0.96 SE, *n* = 61) and from them we calculated parental feeding rate (i.e. the number of parental visits with prey per min). In addition, we determined the total prey richness delivered to the owlets as the number of different prey types detected in the recording of each nest.

### Ethics declaration

Animal data collection complies with the current guidelines and regulations of Spain and the fieldwork was authorized by the regional organism: Consejería de Medio Ambiente y Ordenación del Territorio de la Junta de Andalucía (licence code: P06-RNM-01862). The study protocol was reviewed and approved by the ethical committees of the University of Extremadura (registration number 83/72014) and the CSIC (registration number 013AL01008). Furthermore, all methods are reported in accordance with ARRIVE guidelines.

### Statistical analysis

Our analyses were initially based on a dataset of 132 scops owl nests. However, because some pairs reproduced in multiple years during the study, we randomly selected one reproductive event per pair to avoid pseudo-replication. Therefore, assortative mating was evaluated in 111 pairs (i.e. 222 individuals) over the 10 years of the study.

To verify sexual size dimorphism in our population, we first explored intersexual differences in wing length, bill length, tarsus length, and weight by performing t-tests for independent samples. Females had significantly greater wing length (3.40% larger), bill length (2.68% larger), tarsus length (1.75% larger) and weight (40.18% larger) than males (Table S1). For our analyses we chose wing length as the proxy for body size because it is the most common and standardized measurement of body size in studies of birds^63^. We also analysed sexual size dichromatism by performing a t-test for independent samples on color score.

Then, we analysed the covariation between coloration and body size by performing a linear model (LM, GLM procedure in SAS) with wing length as the dependent variable, and sex, color score and its interaction as predictors.

Later, we investigated assortative mating in size and color by fitting two LMs with wing length and color score of the male, respectively, as dependent variables and the corresponding female traits as predictors. The relative age of the female was included as a covariate in the models to account for the potential effect of breeding experience on mate matching. The LM for plumage color also included the quadratic term of female coloration because previous work in the species has shown that individuals with contrasting coloration might prefer individuals of similar coloration to mate^47^.

To assess the fitness consequences of non-random mating, we first calculated, for each pair, the sum of wing lengths and, separately, the sum of color scores. These metrics were used as indicators of overall pair trait values, with higher values indicating larger overall size and browner overall coloration, respectively. Additionally, to evaluate phenotypic asymmetry within pairs, we used separate approaches for body size and coloration, considering the presence of reversed sexual size dimorphism and the absence of sexual dichromatism in the species. For body size, we first fitted a linear model of wing length as a function of sex and then extracted the residuals for each individual, representing their deviation from the sex-specific mean. The within-pair asymmetry was calculated as the difference between the female and male residuals. This method is suited for sexually dimorphic species because it accounts for basal sexual differences^[see details in 64^. In this context, residuals near 0 indicate pairs in which both individuals are close to the average size expected for their sex (i.e., consistent with the reversed sexual size dimorphism), negative values indicate pairs with relatively similar-sized individuals, and positive values indicate highly dimorphic pairs, with larger than average females and smaller than average males. For coloration, which is not sexually dimorphic in this species (see results), we calculated the absolute difference between the color morph scores of each pair member. This index reflects the degree of chromatic asymmetry between individuals in a pair, with lower values indicating more similar color morphs. This approach allows us to distinguish between the effects of overall trait expression and the asymmetry between pair members. We determined the degree of multicollinearity among the four assortative mating predictors through estimation of VIF (Variance Inflation Factor) and found that predictors do not share a large amount of variance (VIFs lower than 1.5 in all cases). We thus fitted a first generalized linear mixed model (GLMM, GLIMMIX procedure in SAS) with a Poisson error distribution and a log link function with the number of fledglings per nest as the dependent variable and the overall pair value and asymmetry in size and color of the pairs as predictors. The model included the year as a random intercept to account for environmental variability. Additionally, to test whether there were effects of size and color overall pair value and asymmetry on the probability of each chick to fledge (fledging success), we fitted another GLMM with a binomial distribution (successful fledgling = 1, no fledgling = 0) and a logit link function. This model also included brood size as a covariate to account for the potential effects of nest mates and the year and the nest ID as two random intercepts to account for environmental variability and repeated measures within nests.

We subsequently evaluate the effects of size and color assortative mating on quality of the chicks by fitting two linear mixed models (LMMs, MIXED procedure in SAS) with body mass and PHA response of each owlet as dependent variables, respectively. Brood size was also included in all these models as a covariate to control for a possible effect of nest mates. These models included the year and the nest ID as random effects.

Finally, we tested the effects of size and color assortative mating on parental feeding behavior. We fitted a LMM with feeding rates per nest and one GLMM with prey richness (modeled using a Poisson error distribution with a log link function) as the respective response variables. These models included the overall value and asymmetry in size and color of the pair as predictors, and the year as a random intercept.

Overdispersion of statistical models with Poisson error distributions was measured as the ratio of the Pearson χ2 value to its corresponding degrees of freedom n – p.

All analyses were performed in SAS. Sample sizes varied depending on each variable evaluated (for further details, see Supplementary material).

## Electronic supplementary material

Below is the link to the electronic supplementary material and also archived in DIGITAL.CSIC.

Supplementary Material 1

Supplementary Material 2

Supplementary Material 3

Supplementary Material 4

Supplementary Material 5

## Data Availability

Data is provided as supplementary information files.
