# Supplementary material for: Size, not color, drives assortative mating and influences fledging survival, weight and immunity in a polymorphic owl
Source: Sci Rep. 2025 Jun 2;15:19312. doi: 10.1038/s41598-025-04191-1 (PMC12130179; doi:10.1038/s41598-025-04191-1)

**Size, not color, drives assortative mating and influences fledging survival, weight and immunity in a polymorphic owl.**

**Table S1.** Intersexual differences for several phenotypic traits among individuals (F = females, M = males) included in the studied breeding pairs of Scops owls (*Otus scops*). Bold values indicate p values < 0.10.

| **Traits** | *Mean F* | *Mean M* | *n* | *Student t-values* | *df* | *p* |
| --- | --- | --- | --- | --- | --- | --- |
| Wing length | **155.00** | **149.90** | **221** | **8.02** | **219** | **<0.001** |
| Bill length | **9.56** | **9.31** | **222** | **3.53** | **220** | **<0.001** |
| Tarsus length | **27.26** | **26.79** | **221** | **2.69** | **219** | **0.008** |
| Weight | **97.61** | **69.63** | **221** | **26.21** | **219** | **<0.001** |
| Color score | 6.09 | 6.16 | 217 | -0.29 | 215 | 0.77 |

**Table S2.** LMs testing for assortative matting in the scops owl by: 1) wing length, and 2) color score and accounting for the relative age of females. Bold values indicate p values < 0.10.

| **Predictors** |  | **Male’s Trait** | | | | | |
| --- | --- | --- | --- | --- | --- | --- | --- |
|  | *Estimates* | | *Std. error* | *t-value* | *p* | *df* | *R^2^* |
|  | (n = 110) | | **Wing length** | | | | 0.139 |
| (Intercept) | **100.59** | | **14.13** | **7.12** | **<0.001** | **107** |  |
| Female wing length | **0.33** | | **0.09** | **3.64** | **<0.001** | **107** |  |
| Female relative age | **-0.90** | | **0.37** | **-2.42** | **0.017** | **107** |  |
|  | (n = 106) | | **Color score** | | | | 0.061 |
| (Intercept) | 2.28 | | 1.93 | 1.18 | 0.241 | 102 |  |
| Female color score | **1.46** | | **0.66** | **2.23** | **0.028** | **102** |  |
| Female color score^2^ | **-0.13** | | **0.05** | **-2.38** | **0.019** | **102** |  |
| Female relative age | 0.03 | | 0.12 | 0.24 | 0.810 | 102 |  |

**Figure S1.** Relationship between wing length and color morph score of female and male Scops owls (*Otus scops*). Fitted lines are derived from the linear models performed to analyse the covariation between coloration and body size with wing length as the dependent variable, and sex, color score and its interaction as predictors. The shaded areas represent the 95% confidence intervals of the fitted models for males (in blue) and females (in pink). In addition, uncorrected raw data is shown as transparent blue dots (males) and pink triangles (females) in the background. Colors on X axe indicate the color morph of individuals as grey (scores<5.5), intermediate (scores between 5.5 and 7, both included), and brown (scores >7).


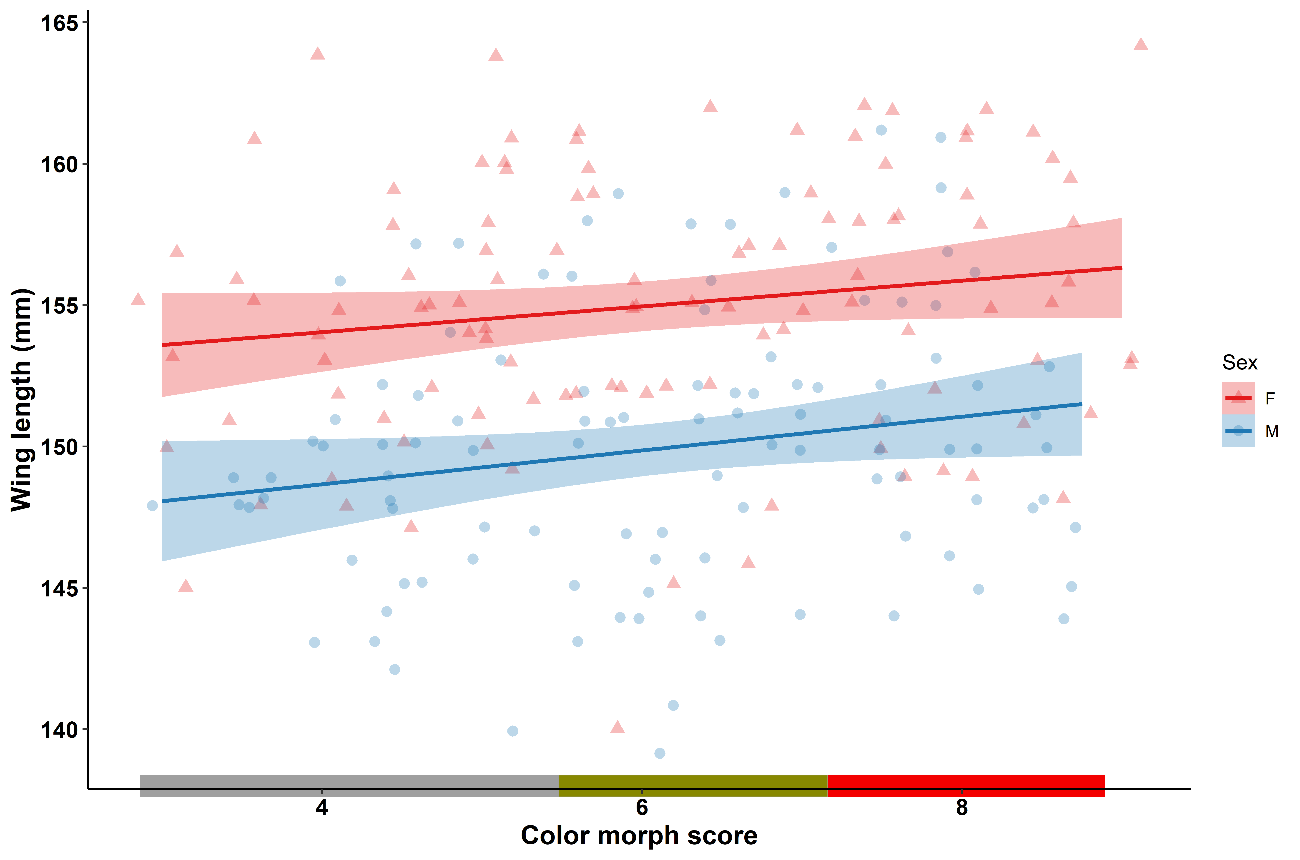

Supplement: Supplementary file 2 — Supplementary Material 2 [file 41598_2025_4191_MOESM2_ESM.docx]
