# Supplementary material for: Size, not color, drives assortative mating and influences fledging survival, weight and immunity in a polymorphic owl
Source: Sci Rep. 2025 Jun 2;15:19312. doi: 10.1038/s41598-025-04191-1 (PMC12130179; doi:10.1038/s41598-025-04191-1)
Supplement: Supplementary file 4 — Supplementary Material 4 [file 41598_2025_4191_MOESM4_ESM.docx]

Codes for statistical analyses to be performed in SAS. The name of the dataset is the name of the excel file including these data that must be imported to SAS.

1. Intersexual differences in wing, tarsus and bil length and weight.

**proc** **ttest** data=Individual_data;

class Sex;

var Wing_length Tarsus_length Bill_length Weight;

**run**;

1. Covariation coloration-body size

**proc** **glm** data=Individual_data;

class Sex;

model Wing_length=Sex|Color_score/ss3 solution;

**run**;

1. Assortative mating by body size

**proc** **glm** data=Data_per_nest;

model Wing_length_Male=Wing_length_Female Relative_age_Female/ss3 solution;

**run**;

1. Assortative mating by plumage coloration

**proc** **glm** data=Data_per_nest;

model Color_score_Male=Color_score_Female Color_score_F2 Relative_age_Female/ss3 solution;

**run**;

1. Fitness consequences of assortative mating
2. On Number of fledglings per nest

**proc** **glimmix** data=Data_per_nest;

class Year;

model N_fledglings = Sum_WING Sum_COLOR Dif_resid_wing Dif_COLOR_FM_abs / dist=p LINK=LOG solution;

random Year;

run;

1. On Fledging success

**proc** **glimmix** data=Data_per_fledging;

class Year Nest;

model Fledging_probability = Sum_WING Sum_COLOR Dif_resid_wing Dif_COLOR_FM_abs Brood_size/ dist=bin LINK=logit solution;

random Year Nest/solution;

run;

1. On Fledging body mass

**proc** **mixed** covtest data=Data_per_fledging;

class Year Nest;

model Fledging_weight= Sum_WING Sum_COLOR Dif_resid_wing Dif_COLOR_FM_abs Brood_size/solution;

random Year Nest/solution;

**run**;

1. On Fledging PHA

**proc** **mixed** covtest data=Data_per_fledging;

class Year Nest;

model Fledging_PHA= Sum_WING Sum_COLOR Dif_resid_wing Dif_COLOR_FM_abs Brood_size/solution;

random Year Nest/solution;

**run**;

1. Feeding efficiency in relation to size and color
2. Feeding rate

**proc** **mixed** covtest data=Data_per_nest;

class Year;

model Feeding_rate= Sum_WING Sum_COLOR Dif_resid_wing Dif_COLOR_FM/solution;

random Year/solution;

**run**;

1. Prey richness

**proc** **glimmix** data=Data_per_nest;

class Year;

model Prey_richness = Sum_WING Sum_COLOR Dif_resid_wing Dif_COLOR_FM / dist=p LINK=LOG solution;

random Year/solution;

run;
